# Health along the corridor: Medical Cooperation and public health development under the China-Pakistan Economic Corridor, 2016-2025

**DOI:** 10.12669/pjms.42.2.14527

**Published:** 2026-02

**Authors:** Qu Qiumei, Lu Pingjia

**Affiliations:** 1 Qu Qiumei Visiting Lecturer, Department of History & Pakistan Studies, University of the Punjab, Lahore, Pakistan; 2 Lu Pingjia Assistant Professor, School of Maxism, Yunnan Technology and Business University, Kunming, China

**Keywords:** Medical Cooperation, Public Health Development, China-Pakistan Economic Corridor, Health Silk Road

## Abstract

The China-Pakistan Economic Corridor (CPEC), as a component of the Belt and Road Initiative, has progressively expanded its scope of cooperation from initial infrastructure and energy projects to encompass health and human development. This study examines the institutional evolution of bilateral health cooperation under the China-Pakistan Health Silk Road (HSR) framework from 2016 to 2025, analyzing the decade-long development of the HSR: deepening collaboration and global health governance, medical infrastructure, capacity building, and technology transfer, alongside socioeconomic and developmental implications. Findings reveal that health cooperation under CPEC pioneers a new paradigm in development diplomacy-interconnectivity extends beyond transport and trade to encompass human security and collective wellbeing. In summary, the future advancement of the ‘Health Corridor’ necessitates overcoming a series of challenges: addressing biosecurity concerns, bridging regional disparities in health standards, and tackling funding shortages and talent gaps in underdeveloped areas along the corridor.

## INTRODUCTION

The China-Pakistan Economic Corridor (CPEC), a key component of China’s Belt and Road Initiative (BRI), is a revolutionary economic and infrastructure project that is changing the geopolitical and economic environment of South Asia. Launched in 2013, the CPEC is a three-thousand-kilometer project that connects Pakistan’s Gwadar Port, a vital hub for international trade and energy routes, with China’s Xinjiang province (China’s State Council Information Office, 2020). While CPEC is frequently referred to as a “game-changer” for national development, it offers Pakistan substantial economic opportunities by addressing long-standing infrastructure deficiencies and energy shortages. For China, it opens up access to the Arabian Sea, improving connectivity to Middle Eastern and African markets.^1^

The CPEC is an economic and development initiative between China and Pakistan, encompassing cooperation in infrastructure, energy, industry, agriculture, science and information technology, alongside the development of Gwadar Port and urban areas, as well as people-to-people exchanges. It aims to foster socio-economic development, promoting prosperity not only for Pakistan and China but for the entire region. The CPEC will enhance regional connectivity, thereby improving trade and economic cooperation across the region.^2^

The Health Silk Road (HSR), as the dedicated component of the Belt and Road Initiative focused on international health cooperation, saw its deployment decisions and implementation over a full five years prior to the outbreak of the COVID-19 pandemic. Marking its tenth anniversary, the HSR has become an integral component of China’s efforts to strengthen global health governance and deepen international public health partnerships. Launched in 2016 as an extension of the Belt and Road Initiative, the HSR aims to promote more equitable access to health resources, advance capacity building, and narrow disparities among participating countries-particularly those in the Global South and ASEAN nations. These regions, characterized by diverse health systems and persistent disparities, represent both key beneficiaries and vital testing grounds for HSR cooperation. Its evolution underscores the growing significance of non-Western and transregional models within international health policy frameworks.^3^

As a branch of the Belt and Road Initiative, the Health Silk Road provides an informational model for Beijing’s strategic planning and its use of specific mechanisms to expand global influence: from establishing healthcare standards to penetrating foreign markets, from shaping international norms to influencing future health governance in third world countries. The Health Silk Road is not a public relations exercise built upon empty slogans. It constitutes a systematic endeavour whose deployment demonstrates the Chinese Party-State apparatus’s capacity to mobilize multiple internal departments and affiliated institutions, while effectively integrating nominally non-governmental or non-state actors to collectively realise its strategic vision. The Health Silk Road embodies the Chinese Party-State’s conception of a future China-led order-one founded upon the rejection of Western value systems and their replacement with alternatives imbued with Chinese essence.^4^

## METHODOLOGY

With an emphasis on its implications for global health governance and partnerships, this policy and practice assessment seeks to examine the HSR’s governance structure, policy procedures, and implementation experience over the last ten years. The approach identifies both successes and difficulties in HSR practice by combining comparative policy assessment and qualitative document analysis.

The Pharmacopoeia of the People’s Republic of China and official policy documents from Chinese government agencies (including the State Council, National Development and Reform Commission, Ministry of Foreign Affairs, and National Health Commission) were among the many reliable sources from which data and materials for this review were gathered. Reports and instructions from international organizations, including the World Health Organization (WHO), World Trade Organization (WTO), Certificate for Documentary Credit Specialists (CDCs), African Union, and Association of South East Asian Nations (ASEAN), were gathered. Additional academic research and policy papers were sourced from organizations such as the Center for Strategic and International Studies (CSIS), Boston University’s Global Development Policy Center, and the ASEAN-China Center. Sources of media coverage included China Daily, China Global Television Network CGTN, and Xinhua News Agency.

Credibility, publication date, and applicability to the HSR’s governance, policy execution, and international collaboration were taken into consideration when choosing the materials. Documents released after the HSR’s 2016 introduction were given special attention.

### Phase-I (2016–2018): Institutional Inception and Policy Embedding Stage:

As China assumes an increasingly significant role in global health, cooperation between the World Health Organization and the Chinese government has grown ever closer and more strategic in nature. The concept of the ‘Health Silk Road’ was first proposed in 2016. In January 2017, coinciding with President Xi Jinping’s historic visit to Geneva, the WHO and China signed a Memorandum of Understanding on health cooperation within the framework of the Belt and Road Initiative. This memorandum outlines a blueprint for strengthening existing cooperation and highlights several specific areas for prioritisation in the coming years, including: (1) Implementation of the 2005 International Health Regulations, public health emergency response, management and capacity building, and the development of emergency medical teams; (2) Health systems and universal health coverage policies; (3) Prevention and control of communicable diseases, including HIV/AIDS, tuberculosis, malaria and schistosomiasis; (4) Prevention and control of non-communicable diseases; (5) Traditional medicine, including Chinese medicine and vaccine prequalification; (6) Capacity building and personnel training; (7) Local production of medicines; (8) Other areas of mutual interest in global health cooperation.^5^

### Phase-II (2019–2021): Mechanism Development and Project Implementation Phase:

The period from 2019 to 2021 marked a pivotal transition for the Health Silk Road initiative within the China-Pakistan Economic Corridor (CPEC) framework, shifting from a policy proposal towards institutionalization and practical implementation. This phase was characterized by the institutional establishment of cooperative mechanisms and the concrete execution of health projects, signifying a turning point in Sino-Pakistani health cooperation from conceptual promotion to systematic implementation.^6^

Against the backdrop of the global COVID-19 pandemic, the pace of health cooperation between China and Pakistan accelerated markedly. Through the signing of multiple Memoranda of Understanding (MoUs) covering infectious disease prevention and control, laboratory capacity building, public health governance, and medical training, the two sides formalized and institutionalized their policy communication channels. Concurrently, the implementation focus of China’s ‘Health Silk Road’ initiative has shifted from macro-strategy to concrete actions. These encompass medical supplies assistance, vaccine collaboration, hospital construction, medical team deployments, and public health training programmes, reflecting a tangible transition from policy advocacy to project execution.^7^

Overall, the period from 2019 to 2021 may be regarded as a dual-track advancement phase for the Health Silk Road within the China-Pakistan framework: on one hand, establishing institutional mechanisms and cooperative frameworks at governmental and organizational levels; on the other, implementing health and public health projects on the ground and demonstrating tangible outcomes. The institutional foundations and project experience accumulated during this phase have laid a solid groundwork for the sustainable and systematic development of future China-Pakistan health cooperation.^8^

### Phase-III (2022–2025): Institutional Deepening and Regional Health Governance Expansion Phase:

In February 2021, the Chinese Centre for Disease Control and Prevention and the Pakistan Branch of the National Institutes of Health signed a memorandum of understanding (Chinese Center for Disease Control and Prevention & National Institute of Health, Pakistan, 2022). This partnership primarily focuses on infectious disease prevention and control, laboratory quality and biosafety, and training in molecular diagnostic technologies. This collaboration helps reduce the risk of cross-border disease transmission, enhances regional capacity to respond to public health emergencies, and ultimately benefits China’s own health security.^9^

In 2024, medical institutions from China and Pakistan signed a Memorandum of Understanding in Kashgar, Xinjiang, China, aimed at deepening bilateral cooperation in the health sector, particularly in medical services, research, and technology. Key topics discussed included international medical collaboration, telemedicine development, infectious disease control, artificial intelligence, the application of big data in healthcare, and the promotion of traditional Chinese medicine.^10^

On January 1^st^ 2022, the Regional Comprehensive Economic Partnership Agreement formally came into effect, further advancing the new development paradigm where the domestic and international dual circulation mutually reinforce each other.^11^

### Medical Infrastructure, Capacity Building, and Technology Transfer:

The case of Pakistan offers rich and multifaceted evidence for understanding the significance of China’s “Health Silk Road”. As a flagship project of China’s Belt and Road Initiative, the China-Pakistan Economic Corridor aims to strengthen economic cooperation and connectivity between China and Pakistan, while promoting regional integration and development.^11^ Against this backdrop, healthcare has emerged as a significant area of cooperation, focusing on various aspects including the construction of hospitals and medical facilities, capacity-building initiatives, and the provision of medical equipment and training. Notable healthcare projects include the Port Authority Hospital in Gwadar, the Medical Centre at the China-Pakistan Gwadar Fajr Colony Secondary School and Vocational Training Centre, Lady Reading Hospital in Peshawar, and the China-Pakistan Joint Laboratory for Infectious Disease Control and Prevention at the National Institute of Health in Islamabad.^12^

Moreover, China has donated medical equipment to Pakistan, including personal protective equipment, ventilators, testing kits, and COVID-19 vaccines. The two nations have also organised training programmes for medical professionals, exchanges of medical experts, and seminars for sharing knowledge and specialized skills. Such cooperation not only contributes to enhancing Pakistan’s healthcare services but also advances China’s broader objectives in economic, diplomatic, reputational, regional stability, and health security spheres.^13^

Medical projects under the China-Pakistan Economic Corridor have created opportunities for Chinese enterprises to expand exports of medical equipment, pharmaceuticals and related services. For instance, Jiangsu Provincial Construction Group secured a multi-million-dollar contract to construct the Port Authority Hospital in Gwadar, Pakistan. These projects have not only secured contracts for Chinese construction firms but also created employment opportunities for Chinese workers, thereby bolstering China’s domestic economy.^14^

Medical cooperation has strengthened bilateral ties between China and Pakistan, deepened friendly relations, and fostered a long-term partnership. Pakistan was among the first nations to receive donated COVID-19 vaccines from China, with 1.2 million doses of the Sinopharm vaccine donated in February 2021 serving as testament to this close relationship. Such medical assistance and healthcare infrastructure development can translate into greater political support for China within international forums, while also fostering cooperation on other strategic initiatives.^15^

### Socioeconomic and Developmental Implications:

Healthcare projects under the China-Pakistan Economic Corridor have fostered Pakistan’s social and economic development, thereby promoting regional stability and security. For instance, in Gwadar, China Overseas Port Holding Company established a girls’ primary school and is currently developing a vocational training institute alongside a 500-bed hospital incorporating a medical center. All these initiatives are funded by grants from the Chinese government.

A healthier population can reduce the likelihood of conflicts, migration and other issues that may destabilize the region, creating a safer environment for China’s own domestic development and international interests. Healthcare project cooperation has enhanced China’s health security by improving disease surveillance, prevention and control in Pakistan. In February 2021, the Chinese Centre for Disease Control and Prevention and the Pakistan branch of the US National Institutes of Health signed a memorandum of understanding.^16^

This partnership primarily focuses on infectious disease prevention and control, laboratory quality and biosafety, and training in molecular diagnostic techniques. This collaboration helps reduce the risk of cross-border disease transmission, enhances the region’s capacity to respond to public health emergencies, and ultimately benefits China’s own health security. The establishment and operation of the China-Pakistan Friendship Hospital have significantly alleviated the region’s scarcity of medical resources, rudimentary healthcare facilities, and limited diagnostic capabilities. With the hospital’s sustained operation, maternal and neonatal mortality rates in the area have shown a downward trend, while immunization coverage has increased. For many years, Sino-Pakistani medical cooperation, as a vital component of the China-Pakistan Economic Corridor collaboration, has consistently warmed the hearts of both nations’ populations. From 22 to 23 January 2025, the emergency project for congenital heart disease treatment in Pakistan was successfully completed. The team from Fuwai Hospital of the Chinese Academy of Medical Sciences, working alongside Pakistani medical staff, successfully performed surgery on eight local children with congenital heart conditions.

There is a need to establish healthcare facilities along CPEC route. Establishment of such healthcare facilities will not only be a health security to goods transporters of the route but also improve tourism along the belt.

Moreover, Comprehensive Primary Health Care (CPHC) will encourage health market in both the countries. For instance, pharmaceutical cooperation between the countries will make cheaper raw materials available for pharmaceutical companies in Pakistan, ensuring cost-effective drug treatments in Pakistan. Moreover, bilateral cooperation through research and joint collaborations will help improve bioengineering, data analytics and rapid flow of information between the countries, coping with health challenges faced by health department of Pakistan.

### The Biosecurity Situation Along the China-Pakistan Economic Corridor is Critical:

Since the 21st century, growing populations and convenient transport systems have heightened the risk of major infectious disease outbreaks, while accelerated globalization and frequent international exchanges have increased the threat of cross-border transmission. Beyond traditional pathogens such as cholera, plague and HIV/AIDS, emerging infectious diseases fuelled by environmental pollution and global climate change continue to emerge. Prior to COVID-19, diseases such as severe acute respiratory syndrome (SARS), highly pathogenic avian influenza (H5N1), and Middle East Respiratory Syndrome (MERS) had already spread extensively along the Belt and Road routes. For instance, South and Southeast Asia exhibit higher infectious disease incidence rates and heavier disease burdens due to high population density and inadequate healthcare infrastructure: South Asia faces severe outbreaks of leprosy, tuberculosis, AIDS, and malaria; while Southeast Asia grapples with significant epidemics of whooping cough, cholera, malaria, and AIDS.

### Significant Disparities Exist in Health Standards Across the China-Pakistan Economic Corridor Region:

The constraints imposed by hard infrastructure resources represent a significant challenge for medical project cooperation within the China-Pakistan Economic Corridor. Underdeveloped regions along the corridor generally suffer from lagging infrastructure, characterised by underdeveloped transport networks, low transport efficiency, inadequate communication facilities, and low telephone and internet penetration rates. In some areas, insufficient power supply further hinders the development of healthcare systems. In certain Pakistani regions, road passenger and freight transport account for 90% and 96% of total transport volumes respectively, yet the road density stands at merely 0.32 kilometers per square kilometer, significantly complicating the transportation of medical supplies. Concurrently, Pakistan’s electricity grid in certain areas is relatively outdated, with comprehensive line losses in transmission and distribution reaching 20%. This results in tight power supply, having led to multiple large-scale blackouts. Power shortages and supply instability severely disrupt the operation of medical equipment and the conduct of routine medical work.

### Funding Shortages and Talent Scarcity in Underdeveloped Regions Along the China-Pakistan Economic Corridor:

Funding and talent constitute the two fundamental pillars for building the ‘Health Silk Road’. The underdeveloped economic conditions in less-developed regions along the corridor constrain investment in construction funds. Whether it concerns enhancing basic healthcare standards or strengthening medical emergency management capabilities, stable and sustained diversified funding is urgently required. Concurrently, there exists a significant shortage of multidisciplinary medical professionals. Take the China-Pakistan Medical Emergency Centre in Gwadar Port as an example: this facility requires not only specialised medical personnel but also public health policy officers and Red Cross liaison officers. Due to underdeveloped economies, some Belt and Road partner nations face substandard educational and healthcare environments, hindering both the cultivation of local talent and the attraction of international specialists. This dual constraint severely limits progress in elevating medical standards.

Investment in healthcare infrastructure under the HSR initiative typically relies on loans from Chinese policy banks, multilateral development banks, or other international financial institutions. This financing approach has raised concerns regarding the debt sustainability of participating countries, particularly those with high debt levels or limited debt servicing capacity.^4^ Unsustainable debt levels may lead to economic instability, fiscal constraints and reduced policy space for partner countries, thereby potentially undermining the long-term benefits of HSR projects. Stakeholders must carefully assess and manage debt risks associated with high-speed rail investments to ensure the financial sustainability of these projects.^16^

### Conclusions and Policy Recommendations:

Medical cooperation and public health initiatives under the ‘Health Silk Road’ framework evolved along the CPEC corridor from 2016 to 2025. The Belt and Road Initiative’s first focus was on infrastructure, energy projects, trade facilitation, and financial cooperation. Over time, it evolved to improving the lives of people in the countries that took part, as well as their health care and education. Through initiatives such as the China-Pakistan Friendship Hospital, joint laboratories, vaccination programmes, and medical education projects, CPEC has integrated people’s welfare into its interconnected infrastructure framework.

### Phase-I (2016–2018):

Institutional inception and policy embedding phase; *Phase-II (2019–2021):* Mechanism establishment and project implementation phase; *Phase-III (2022–2025):* Institutional Deepening and Regional Health Governance Expansion. This evolutionary trajectory has reinforced the shift in China-Pakistan cooperation from infrastructure to healthcare, fostering not only investment confidence between the two nations but also driving their socio-economic development through the transfer of medical technologies.

To ensure the steady and far-reaching development of the ‘Health Silk Road’ initiative within the China-Pakistan Economic Corridor, policy recommendations should be formulated across four key areas:

Strengthen top-level design to establish a high-quality ‘Health Silk Road’ brand and generate brand effects. The construction of the ‘Health Silk Road’ requires concerted efforts from governments, enterprises, and citizens, each fulfilling their respective roles and collaborating closely. It is recommended that coordinated planning be undertaken at the institutional level, formulating comprehensive and systematic cooperation plans from a strategic perspective to guide specific collaborative initiatives. Tailor approaches to national and regional contexts, fully accounting for the differences between China and Pakistan. For emerging markets and developing economies, prioritize infrastructure development closely linked to life and health, alongside prevention and control of major preventable infectious diseases. For developed economies, actively promote exchanges in traditional medicine and cutting-edge technologies within the life and health sector.

Leverage the pioneering and exemplary role of institutional and mechanism innovation, strengthen regional guidance, and encourage pragmatic cooperation. At the national level, China has established bilateral or multilateral health cooperation relationships with numerous Belt and Road partner countries, establishing high-level cooperative mechanisms. However, the concrete implementation of such cooperation relies upon micro-level entities. At the regional level, relevant exchange and cooperation platforms remain relatively underdeveloped, necessitating supportive and incentive policies to guide efforts. This will encourage localities to fully leverage their resource endowments in establishing diverse cooperative relationships, thereby advancing the development of pragmatic health cooperation projects.

Advance the development of traditional medicine in all respects, deepen exchanges and cooperation in traditional Chinese medicine, and accelerate the pace of traditional Chinese medicine’s internationalization. Traditional Chinese Medicine (TCM) constitutes a vital component of China’s healthcare system. In advancing the ‘Health Silk Road’, its unique strengths in preventive healthcare and treating major diseases should be fully leveraged. We shall actively establish overseas TCM centres and international scientific cooperation bases along the Belt and Road, dispatch TCM medical teams for overseas aid, and enhance understanding and cultural promotion of the holistic life-cycle health philosophy of preventive healthcare. These efforts will contribute to the sustainable development of healthcare systems in both China and Pakistan.

Recognizing the opportunities presented by digitalization in global healthcare and life sciences, we must seize this momentum to leapfrog ahead and comprehensively advance the digital infrastructure of the ‘Health Silk Road’. Presently, the concept of ‘digital health’ has garnered significant investor interest in capital markets, with data-driven digital transformation, wearable devices, and telemedicine all paving the way for digital healthcare development. The China-Pakistan Economic Corridor possesses inherent advantages for developing digital health, including a large population base, high healthcare demand, and relatively underdeveloped healthcare infrastructure. Technological advancement is a double-edged sword; if the conveniences afforded by information technology can be harnessed while effectively safeguarding privacy, it holds promise for advancing the overall health of citizens in both China and Pakistan through digitalization and informatization.
